# Phylogenetic analysis of ionotropic L-glutamate receptor genes in the Bilateria, with special notes on *Aplysia californica*

**DOI:** 10.1186/s12862-016-0871-1

**Published:** 2017-01-11

**Authors:** Justin B. Greer, Sawsan Khuri, Lynne A. Fieber

**Affiliations:** 1 Department of Marine Biology and Ecology, Rosenstiel School of Marine and Atmospheric Science, University of Miami, 4600 Rickenbacker Cswy, Miami, FL 33149 USA; 2 Center for Computational Science, University of Miami, 1320 S. Dixie Highway, Coral Gables, FL 33146 USA; 3 Department of Computer Science, University of Miami, P.O. Box 248154, Coral Gables, FL 33124 USA

**Keywords:** Kainate, Real-time PCR, L-glutamate, Nervous system

## Abstract

**Background:**

The neurotransmitter L-Glutamate (L-Glu) acting at ionotropic L-Glu receptors (iGluR) conveys fast excitatory signal transmission in the nervous systems of all animals. iGluR-dependent neurotransmission is a key component of the synaptic plasticity that underlies learning and memory. During learning, two subtypes of iGluR, α-Amino-3-hydroxy-5-methyl-4-isoxazolepropionic acid receptors (AMPAR) and N-methyl-D-aspartate receptors (NMDAR), are dynamically regulated postsynaptically in vertebrates. Invertebrate organisms such as *Aplysia californica* (*Aplysia*) are well-studied models for iGluR-mediated function, yet no studies to date have analyzed the evolutionary relationships between iGluR genes in these species and those in vertebrates, to identify genes that may mediate plasticity. We conducted a thorough phylogenetic analysis spanning Bilateria to elucidate these relationships. The expression status of iGluR genes in the *Aplysia* nervous system was also examined.

**Results:**

Our analysis shows that ancestral genes for both NMDAR and AMPAR subtypes were present in the common bilaterian ancestor. NMDAR genes show very high conservation in motifs responsible for forming the conductance pore of the ion channel. The number of NMDAR subunits is greater in vertebrates due to an increased number of splice variants and an increased number of genes, likely due to gene duplication events. AMPAR subunits form an orthologous group, and there is high variability in the number of AMPAR genes in each species due to extensive taxon specific gene gain and loss. qPCR results show that all 12 *Aplysia* iGluR subunits are expressed in all nervous system ganglia.

**Conclusions:**

Orthologous NMDAR subunits in all species studied suggests conserved function across Bilateria, and potentially a conserved mechanism of neuroplasticity and learning. Vertebrates display an increased number of NMDAR genes and splice variants, which may play a role in their greater diversity of physiological responses. Extensive gene gain and loss of AMPAR genes may result in different physiological properties that are taxon specific. Our results suggest a significant role for L-Glu mediated responses throughout the *Aplysia* nervous system, consistent with L-Glu’s role as the primary excitatory neurotransmitter.

**Electronic supplementary material:**

The online version of this article (doi:10.1186/s12862-016-0871-1) contains supplementary material, which is available to authorized users.

## Background

L-Glu is the most abundant neurotransmitter in the vertebrate brain [1], and exerts most of its effects by binding to different postsynaptic ligand-gated receptors. L-Glu receptors are classified into two types, ionotropic and metabotropic receptors [2]. Metabotropic L-Glu receptors (mGluR) are G-protein-coupled receptors in which binding of L-Glu activates intracellular cascades and modification of intracellular proteins. Ionotropic L-Glu receptors (iGluR) convey the majority of fast excitatory signal transmission and have been implicated in most aspects of central nervous system (CNS) development and function [3]. The binding of L-Glu to iGluR opens transmembrane ion channels that allow ions to cross the plasma membrane leading to depolarization of the postsynaptic cell and triggering of action potentials, thereby transmitting synaptic information. iGluR play important roles in synaptic plasticity, which is the ability of a synapse to strengthen or weaken its interactions with others over time in response to changes in activity. This feature of iGluR is believed to be a key mechanism underlying learning and memory [4].

Several features of iGluR in vertebrates have been revealed using model systems such as rats and mice. Vertebrate iGluR are divided into three subtypes according to selective agonists: N-methyl-D-aspartate receptors (NMDAR), kainate, and α-Amino-3-hydroxy-5-methyl-4-isoxazolepropionic acid receptors (AMPAR). Each subtype of iGluR is composed of four subunits that form a dimer of dimers to create the functional protein [5]. In a phylogenetic analysis of human, rat, and mouse iGluR subunits, each of the three different subtypes formed a monophyletic clade, with functional iGluR proteins made only with subunits within each individual clade [6]. Overall amino acid identity of iGluR subunits across the three subtypes in mammals is only 20–30%, but all subtypes contain common structural features that place them together into a single large superfamily [7]. A fourth iGluR subtype, called delta receptors, show low sequence similarity with other iGluR and do not open ion channels, but are believed to bind D-serine and glycine [8].

Invertebrate model species such as *Aplysia californica* (*Aplysia*), *Drosophila melanogaster* (*Drosophila*), and *Caenorhabditis elegans* (*C. elegans*) have been used extensively in studies of iGluR mediated transmission in the nervous system since the 1960’s due to several distinct advantages over vertebrate models [9]. Their nervous systems contain 302–135,000 neurons, compared to 1 × 10^11^ in the human brain [10–12], and simple neuronal circuits underlying various behaviors have been described [13–16]. There is ample evidence suggesting that L-Glu is the major neurotransmitter in many neural circuits in these species [17–20]. These advantages have resulted in widespread use of *Aplysia, Drosophila, C. elegans*, and other models to study iGluR mediated synaptic plasticity, because learned behaviors can be correlated with both molecular and physiological synaptic changes between sensory- and motoneurons.

Physiologically, invertebrate model organisms have greatly enhanced our understanding of L-Glu mediated synaptic plasticity. Many plasticity related changes characterized in invertebrate models subsequently have been demonstrated to occur in the more complex vertebrate hippocampus [21]. For example, AMPAR and NMDAR have been implicated in learning and memory in vertebrates, *Aplysia*, and *D. malanogaster* [22–24]. Despite the physiological use of invertebrate model species for iGluR-mediated responses, an outstanding question remains as to which iGluR genes are likely to play the functional role of vertebrate NMDAR and AMPAR during plasticity.

To address this question we have conducted a phylogenetic analysis spanning all three Bilateria superclades in order to identify iGluR genes in invertebrate model species that are orthologous with vertebrate iGluR, and thus more likely to be functionally similar. Bilateria are organized into three superclades based on embryology, morphology and molecular data: Ecdysozoa (including arthropods like *D. melanogaster*, nematodes like *C. elegans*), Lophotrochozoa (including molluscs such as *Aplysia*), and Deuterostomia (including chordates like rats, mice, humans) [25]. Lophotrochozoa and Ecdysozoa are sister-clades that together form the Protostomia [26, 27]. Most studies support the monophyly of these three superclades, and thus a common ancestor for all Bilateria. Several recent studies support a monophyletic grouping of Deuterostomia, with Protostomia as a sister clade [28].

Identification of Protostomia iGluR genes homologous to vertebrate NMDAR and AMPAR subunits can allow for predictions of subunits that may be involved in observed synaptic plasticity. This analysis can also add useful information to our poor understanding of subtype-specific agonists in protostomes [29–31].

*Aplysia* is a model organism with a long history of studies of iGluR-mediated nervous system function, in particular for learning and memory paradigms [9, 32–34]. *Aplysia* NMDAR subunits have been shown to be expressed throughout the nervous system [35], however most other *Aplysia* iGluR have been identified through similarity to sequences of other species, and their *in vivo* expression patterns are unknown. Subunits within each iGluR subtype form a monophyletic clade in vertebrates, and complete receptors can only be formed with subunits within each of these clades. An *Aplysia*-only phylogeny was built to identify subunits that form monophyletic clades, and thus may form functional receptors.

In this study we clarified the evolution of bilaterian iGluR using phylogenetic analysis, investigated iGluR genes in *Aplysia*, and determined expression levels in ganglia of the *Aplysia* nervous system to place this model into an appropriate context with other iGluR model species.

## Methods

### Phylogenetic analysis

iGluR sequences for phylogenetic analysis were obtained from NCBI for all species except *Branchiostoma belcheri* (lancelet), which were obtained from the Chinese Lancelet Genome Project (<genome.bucm.edu.cn/lancelet>). Sequences of all identified canonical iGluR proteins were aligned in MEGA7 [36] using the MUSCLE (multiple sequence comparison by log-expectation) multiple aligner algorithm with default parameters [37]. Poorly aligned regions of the alignment were removed using trimAl [38] with the –automated1 option to heuristically determine optimal trimming of the alignment, resulting in 473 positions used for phylogenetic analysis. Unrooted phylogenetic trees were then constructed in MEGA7 using maximum likelihood and 1000 bootstrap replicates with the LG amino acid substitution model [39] with gamma distributed rates and five rate categories. The initial tree was obtained by applying the Neighbor-Joining algorithm to a matrix of pairwise distances estimated using a JTT model.

Subtype specific trees were built to further clarify the relationships within orthologous groups using the same parameters as the full phylogeny, except 500 bootstrap replicates were performed. These same parameters were also used to build the *Aplysia*-only iGluR tree. Trees were visualized using FigTree (v1.4.2) [40]. Protein accessions for all sequences can be found in Additional file 1.

### Identification of iGluR genes in *Aplysia*

Previously described iGluR in both *Aplysia* and chordates were obtained from the NCBI database, and analyzed using tools at the SMART [41] and Interpro [42] protein databases for potential binding sites and transmembrane domains to identify all iGluR subunits in *Aplysia*. These sites are likely to be highly conserved to maintain L-Glu activation, thus their sequences were extensively searched in both the *Aplysia* published genome (NCBI) and the freely accessible *Aplysia* transcriptome database (<http://www.aplysiagenetools.org/>). Candidate genes were then translated to protein sequences and run through a BLAST search as well as scanned in Interpro for verification. Genes were confirmed to be in the *Aplysia* transcriptome by PCR amplification from cDNA of the abdominal ganglia and cloned in a TA vector (Invitrogen) and subsequently sequenced.

### Hydrophobicity analysis

The Kite/Doolittle hydrophobicity scale was used to determine the hydrophobicity of each amino acid for the last 450 amino acids in both human and *Aplysia* Grin1 subunits, which contains the ligand binding, transmembrane, and intracellular C-terminal domains. For representative AMPAR and kainate receptor subunits only transmembrane domains were analyzed due to lack of agonist-binding site identification in *Aplysia*.

### mRNA extraction and quantification of iGluR gene expression in the nervous system

To describe quantitative expression of identified iGluR in *Aplysia* nervous system ganglia, six sexually mature *Aplysia californica* from a single egg mass of wild-caught animals were obtained from the National Resource for Aplysia at the University of Miami. Animals were anesthetized in a solution of 50:50 isotonic MgCl_2_:Artificial sea water (ASW). All ganglia were removed, immediately rinsed in ASW, and placed in Trizol (Invitrogen). Tissues were ground in a bead homogenizer to break cells out of the sheath prior to RNA extraction. Both hemiganglia for each tissue from each animal were pooled into a single sample.

Total RNA was extracted following the manufacturer’s protocol and samples were treated with DNAse to remove any contaminating DNA. RNA quantities were determined using a Nanodrop (Model ND-1000), and samples were stored at -80 °C until further processing. 100 ng of RNA was reverse transcribed into cDNA using the SuperScript III First-Strand Synthesis System (Invitrogen). After dilution of the cDNA (1:5 with H_2_O) messenger RNA (mRNA) copy number was determined using qPCR on a Stratagene Mx3005P with SYBR Green master mix and the equivalent of 2 ng of starting RNA per well.

Primer pairs were designed for each of the 13 iGluR identified in *Aplysia* to detect expression levels with quantitative real-time PCR (see Additional file 2 for primers). Efficiencies of each primer pair was tested by generating standard curves based on regression analyses of the Ct and the log value of 10-time dilution of each target gene for each primer pair. All primers used had efficiencies between 0.9 and 1.1. mRNA copies were then calculated using standard curves and the average of duplicate cycle threshold (Ct) values.

### Southern blot

Southern blotting from agarose gels was done onto Hybond-N + membrane (Amersham) in sodium saline citrate buffer (10 × SSC; 1.5 M NaCl; 0.15 M Na citrate pH 7.0) by capillary action. Hybridization for high stringency blots was conducted in 30% formamide, 5× SSC, 1× Denhardt’s, 0.2% sodium dodecyl sulphate (SDS), 10% Dextran sulphate, 20 mM sodium phosphate, pH 6.8 and at 42 °C. Final washes for high stringency were in 0.1 × SSC, 0.1% SDS at 65 °C. Each lane contained 4 μg of DNA and was cut with one of the following enzymes: EcoR1, HindIII, BamH1, and PstI. The probe was labeled using a random primed DNA labeling kit with [γ-^32^P] dCTP.

## Results

### Phylogenetic analysis of Bilaterian iGluR subunits

**Fig. 1:**
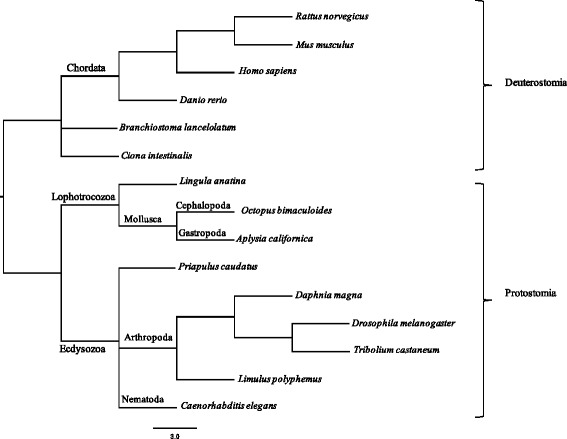
Species tree. Tree of the evolutionary relationships between species used in this study. The tree was built using the NCBI taxonomy browser [80]

To investigate the evolutionary relationships of iGluR across the Bilateria, phylogenetic analysis was conducted on full-length protein sequences of all NMDAR, AMPAR, kainate receptor, and delta receptor subunits. Sequences were included from the three major bilaterian lineages: Deuterostomia (*Homo sapiens*, *Mus musculus, Rattus norvegicus, Danio rerio*, *Branchiostoma lanceolatum*, *Ciona intestinalis*), Ecdysozoa (*Limulus polyphemus*, *Priapulus caudatus*, *Drosophila melanogaster*, *Caenorhabditis elegans*, *Daphnia magna*, *Tribolium castaneum*), and Lophotrochozoa (*Aplysia californica*, *Octopus bimaculoides*, *Lingula anatina*). Additional file 3 contains the multiple sequence alignment of all proteins. The species tree showing the evolutionary relationships among the species is shown in Fig. 1.

**Fig. 2:**
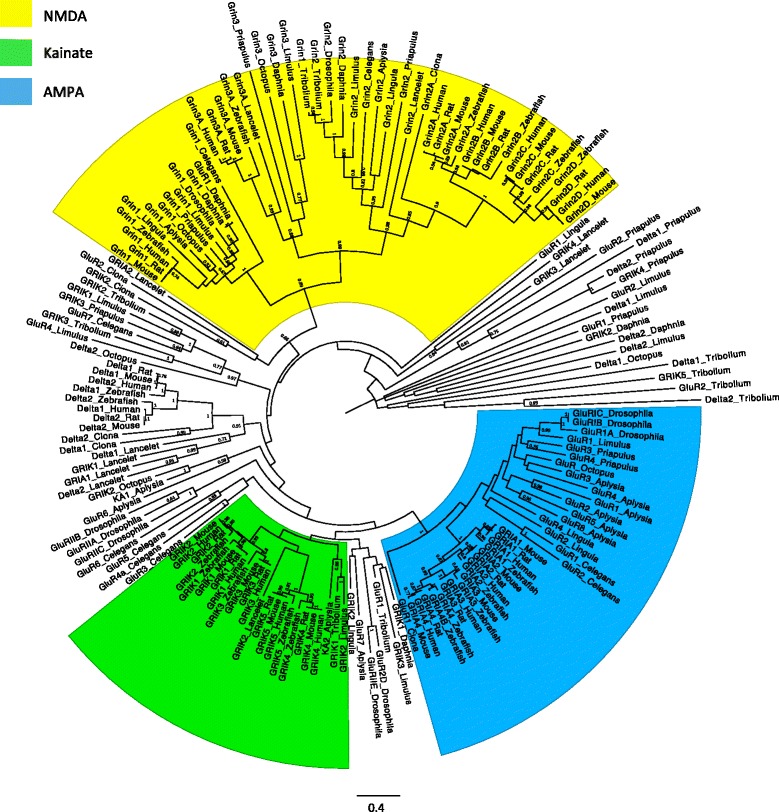
Unrooted bootstrap consensus phylogenetic tree. iGluR protein sequences for six deuterostomes and nine protostomes with iGluR information available were aligned using MUSCLE in MEGA7 and the tree was constructed using maximum likelihood and 1000 bootstrap replicates. iGluR subtypes are indicated by different colors. Numbers indicate bootstrap support and are displayed for boostrap values >0.6. The scale bar represents 0.4 substitutions per site. All species contain homologs for Grin1 and Grin2 subunits. An ancestral AMPAR and kainate receptor gene was present in the common bilaterian ancestor, but in the case of the kainate receptor, the monophyly is only weakly supported by bootstrap values

The phylogenetic tree of bilaterian iGluR is presented in Fig. 2. Identification of iGluR genes as kainate receptor or AMPAR subtypes is unclear for many protostomes, thus these genes are currently named as “GluR” genes without an AMPA or kainate designation. Deuterostome iGluR genes are named Grin (Glutamate Receptor Ionotropic NMDA), GRIA (Glutamate Receptor Ionotropic AMPA), or GRIK (Glutamate Receptor Ionotropic Kainate) based on selective agonists. Canonical sequences, as determined by Uniprot, were used for genes with more than 1 splice variant. Some deep branches of the tree were poorly supported by bootstrapping due to high divergence of sequences between subtypes and large evolutionary distance between species. However, most orthologous groups were well resolved with strong bootstrap support. Based on the phylogeny of the iGluR proteins each protostome subunit was classified as NMDAR, AMPAR, kainate receptor or as orphan genes that do not show a clear relationship with vertebrate subtypes (Table 1).

**Table 1:** Placement of Protostomia iGluR into subtypes based on phylogenies

Species	AMPA	Kainate	NMDA	Orphans
*Aplysia californica*	GluR1 GluR2 GluR3 GluR4 GluR5 GluR8	GluR7 KA2	Grin1 Grin2	KA1 GluR6
*Caenorhabditis elegans*	GluR1 GluR2	none	Grin1 Grin2	GluR3 GluR4a GluR5 GluR6 GluR7
*Drosophila melanogaster*	GluR1A GluR1B GluR1C	GluR2D GluRIIE	Grin1 Grin2	GluRIIA GluRIIB GluRIIC
*Daphnia magna*	none	GRIK1	Grin1 GluR1 Grin2 Grin3	GRIK2
*Limulus polyphemus*	GluR1	GRIK2 GRIK3	Grin1 Grin2 Grin3	GRIK1 GluR4 GluR2
*Lingula anatina*	GluR2 GluR4	GRIK2	Grin1 Grin2	GluR1
*Octopus bimaculoides*	GluR	none	Grin1 Grin3	GRIK2
*Priapulus caudatus*	GluR3 GluR4	none	Grin1 Grin2 Grin3	GRIK3 GluR1 GluR2 GRIK4
*Tribolium castaneum*	none	GRIK1 GluR1	Grin1 Grin2	GRIK2 GRIK3 GRIK5 GluR2

iGluR genes in each protostome species were categorized into subtypes based on their phylogenetic relationship with chordate iGluR genes. Many protostome iGluR genes do not have a clear relationship with chordate subtypes and are thus identified as orphan receptors. Protostome orphan receptors are divergent from chordate genes and thus unlikely to perform the same subtype specific functions

NMDAR genes form three orthologous groups corresponding to Grin1, Grin2, and Grin3 subunits, providing evidence that all three ancestral Grin subunits were present in the most recent bilaterian ancestor (Fig. 2). This is a unique feature of NMDAR: they are the only iGluR subtype with more than one orthologous copy present before the divergence of protostomes and deuterostomes. In the orthologous group Grin2 each protostome and basal deuterostome has only one ortholog of Grin2, but vertebrates have four Grin2 genes. Vertebrate Grin2 genes form a highly discrete clade, with four paralogous copies that arose early in the vertebrate lineage. The Grin2 paralogs in vertebrates are likely best explained by the 2R hypothesis, which postulates that two rounds of whole genome duplication occurred early in the vertebrate lineage after their split from tunicates [43, 44]. Thus, the 2R hypothesis predicts that vertebrates are expected to have four copies of each gene in comparison to one copy in invertebrates. In the case of Grin2 all four paralogs have been retained in all vertebrates used in this study.

In contrast, both in Grin1 and Grin3 there is only a single orthologous group of vertebrates present in the tree. Therefore, in both cases only one of the four that originated during the 2R genome duplications remained active, whereas three of them have been lost early in vertebrate evolution. Despite large evolutionary distances among the studied species, all three Grin orthologs are highly conserved suggesting that they are under high functional constraints slowing their divergence.

For AMPAR genes there is a monophyletic clade of all vertebrate AMPAR sequences and several orthologous protostome genes, pointing to an AMPAR gene copy present in the common bilaterian ancestor (Fig. 2). The number of AMPAR genes in protostome species is highly variable and appears to be species or taxon specific. For example, in the Lophotrochozoa, *Aplysia* has 6 paralogous AMPAR genes, *Octopus* has 1, and *Lingula* has 2. Ecdysozoan species have 2–3 genes in this orthogroup, except *Daphnia* and *Tribolium*, which do not have any genes in the AMPAR orthogroup.

Most AMPAR genes of protostome species are most closely related to each other (inparalogs) rather than to AMPAR genes, with *Lingula* as the exception. This implies that gene duplications occurred after the divergence of each lineage in this study, suggesting that there has been extensive gene gain and loss that has acted independently in each protostome taxon. This appears particularly true in *Aplysia*. Six AMPAR genes in *Aplysia* and 1–2 in other lophotrocozoans, including *Octopus,* suggests several gene duplication events occurred within the Gastropoda lineage. The sampled vertebrates have consistent with the 2R genome duplication scenario all four AMPAR genes, in comparison to one AMPAR ortholog in the common bilaterian ancestor.

Kainate receptor genes are the only subtype in the tree in which Protostomia and Deuterostomia genes do not form a strongly supported monophyletic clade. Only three protostome genes form an orthologous group with chordate kainate receptor subunits: *Aplysia* KA2, *Tribolium* GRIK1, and *Limulus* GRIK2. Identification of kainate receptor genes is unclear for many Ecdysozoan species due to extensive divergence of these subunits from Deuterostomia. Many predicted kainate receptor genes from *Limulus*, *Priapulus*, and *Tribolium* form an independent clade without a clear relationship to chordate kainate receptor genes (to the left of the tree, Fig. 2). This suggests they have a slightly different function, or that they work through a different mechanism than the kainate genes that have been studied. Additionally, *Priapulus*, *Limulus*, *Daphnia*, and *Tribolium* have several genes that appear to be very distant to all other genes in the tree (to the right of the tree, Fig. 2).

Within vertebrates there are two ancient paralogs with a total of five kainate genes. The three paralogs GRIK_A1-3 form one orthologous type and the two paralogs GRIK_B4-5 form together with the lancelet GRIK2 sequence the second type. Therefore, in comparison to the four paralogs expected under the 2R duplications, GRIK_A1-3 and GRIK_B4-5 were respectively losing one and two paralogs. In contrast to Grin and AMPAR genes, extensive divergence of kainate receptor genes in Deuterostomia and Protostomia suggests that they are the least conserved iGluR subtype across Bilateria.

Orthologous groups of each iGluR subtype were further analyzed in the attempt to obtain a better resolution of the evolutionary relationships in these parts of the tree (see Additional file 3 for alignments, Additional file 4 for trees). In this analysis the relationship between protostome and chordate iGluR were the same as in the full phylogeny, providing further support for the relationships found in the full phylogeny.

An additional phylogenetic tree was built using only *Aplysia* iGluR to search for subunits that form monophyletic clades and thus may form complete receptors (See Additional file 3 for alignment, Additional file 4 for tree). The *Aplysia*-only tree confirms the findings of the full phylogeny, with subunits corresponding to currently predicted NMDAR and AMPAR subunits forming monophyletic clades, while the four predicted kainate receptor subunits do not show a monophyletic relationship.

### Number of iGluR genes in *Aplysia* and other Bilaterians

Through genomic searches, the number of iGluR genes identified in chordates was greater than the number of genes in any protostome (Table 2), although it must be noted that for some protostome species the number of iGluR genes may not be accurate due to limited availability of genomic information and annotation. As described in the phylogenetic analysis, extensive gene gain and loss of AMPAR genes in protostomes has also contributed to the variable number of iGluR genes in different protostome species. The increased number of genes in chordates is likely due to retention of many paralogs after 2R genome duplications. We found that in both Grin1 and Grin3 orthologous groups three of the four paralogous genes from the 2R genome duplications have not been retained.

**Table 2:** Number of iGluR genes in the protostomes and deuterostomes

Species	Total iGluR genes	Species	Total iGluR genes
*Homo sapiens*	14	*Caenorhabditis elegans*	9
*Mus musculus*	14	*Ciona intestinalis*	6
*Rattus norvegicus*	14	*Daphnia magna*	7
*Danio rerio*	14	*Limulus Polyphemus*	11
*Aplysia california*	12	*Priapulus caudatus*	11
*Lingula anatina*	6	*Tribolium castaneum*	10
*Octopus bimaculoides*	6	*Branchiostoma belcheri*	10
*Drosophila melanogaster*	10		

**Fig. 3:**
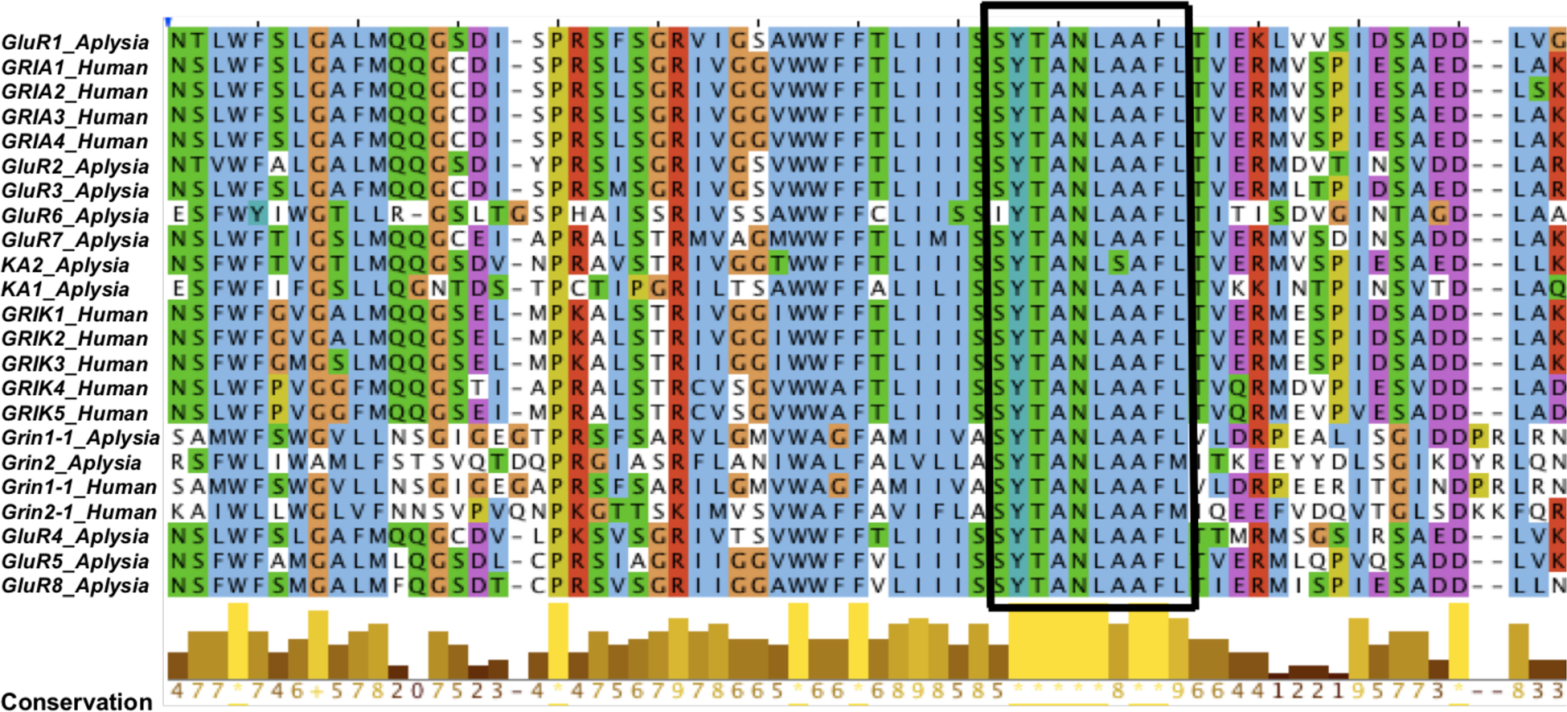
Conserved motifs in *Aplysia* and *H. sapiens*. Boxed region denotes SYTANLAAFL motif vital for formation of the channel pore and activation gate, and in all subunits this high selective pressure has resulted in highly conserved amino acid sequences over large evolutionary distances. This motif is also conserved in all other chordates in this study

Genomic searches using SMART [45] and InterPro tools [46] revealed 12 unique iGluR genes and one splice variant of Grin1 in *Aplysia*. The translated *Aplysia* iGluR sequences showed a highly conserved SYTANLAAF motif [47] near the second transmembrane loop, which was used to identify them as iGluR (Fig. 3). This motif contributes to formation of the channel outer pore and its activation gate [48], and amino acid substitutions in this motif are reported to alter channel gating and permeability [49, 50]. *Aplysia* showed a greater number of iGluR subunits compared to other protostomes, primarily due to several gene duplications of AMPAR genes, as discussed above. cDNAs corresponding to all 12 *Aplysia* iGluR were isolated from the nervous system and confirmed to be transcribed *in vivo.*

### Sequence similarity and conserved domains of Grin1 in *Aplysia* and vertebrates

Conserved domains of Grin1 proteins in *Aplysia* and the vertebrates studied were analyzed to elucidate whether similarities in these parts of the sequences might predict conservation of function. The first ~400 amino acids of NMDAR proteins, known as the N-terminal domain (NTD), show high sequence divergence, with only 19% sequence identity in this region within a species. Mutant Grin2 subunits lacking the entire NTD were shown to assemble into receptors functionally similar to complete receptors, thus it is not surprising that this region showed low sequence similarity between species [51, 52]. Comparatively, agonist binding domains (ABDs) and transmembrane domains (TMDs) must show greater conservation to maintain iGluR functionality, and indeed, within a species these regions show 63 and 73% identity, respectively [53]. In comparing the Grin1 subunits of *H. sapiens* and *Aplysia* we found 66% sequence identity between these subunits after removal of the NTD, similar to the sequence similarity of ABDs and TMDs within a species.

The three TMDs of Grin1 together form the iGluR ion channel and these regions show very high protein sequence similarity throughout the Bilateria. However, *Aplysia* sequences consistently showed greater sequence similarity to *H. sapiens* than did either *D. melanogaster* or *C. elegans* (Table 3). The TMDs of the vertebrate genes compared had close to 100% conservation, on average, and exhibited much greater amino acid similarity than the rest of the protein, suggesting high selective pressure on these sites to maintain function.

**Table 3:** Sequence similarities in transmembrane domains of Grin1 between different protostomes and *H. sapiens*

Species	TMD1	TMD2	TMD3
*A. californica*	85%	100%	87%
*D. melanogaster*	75%	100%	61%
*C. elegans*	65%	87%	48%

Compared to *H. sapiens*, *Aplysia* had fewer amino acid substitutions in transmembrane domains (TMDs) than did *D. melanogaster* and *C. elegans*. This points to a higher likelihood of conserved channel function between vertebrates and *Aplysia*

**Fig. 4:**
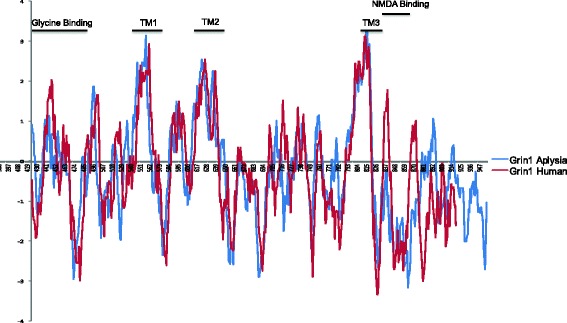
Hydrophobicity plot of Grin1 in *H. sapiens* and *Aplysia*. Kite/Doolittle scale was used to determine the hydrophobicity of each amino acid for the last 450 amino acids, containing the ligand binding domain, TMD, and intracellular C-terminal domain. Despite large evolutionary distance between *Aplysia* and *H. sapiens*, only substitutions with similar hydrophobicity are tolerated in TMDs to maintain proper folding of the ion channel. Predicted NMDA and glycine binding sites show many more substitutions that result in changes in hydrophobicity

The hydrophobicity of amino acids in a protein influences the folding and structure of the molecule, and protein regions with similar hydrophobicity profiles are predicted to maintain structural stability. TMDs are well conserved, but substitutions that result in different amino acids occur, as shown in Table 3. A plot of the hydrophobicity of the canonical Grin1 sequences in *Aplysia* and *H. sapiens* shows that amino acid substitutions in the TMD’s have been tolerated only when the substituted residue has a similar hydrophobicity, and thus is predicted not to significantly alter protein folding (Fig. 4). This result suggests that the structure and function of the transmembrane domains and ion channel have been maintained. Conversely, protein sequences for the glycine and NMDA binding sites show high sequence divergence and numerous changes in hydrophobicity compared to *H. sapiens* sequences. Hydrophobicity plays a crucial role in receptor binding domains [54], and this lack of conservation may reflect the diminished role for glycine as a co-agonist in *Aplysia* NMDAR [55].

Representative hydrophobicity plots of AMPAR and kainate receptor subunits can be found in Additional file 4. For both AMPAR and kainate receptor subunits, hydrophobicity of the TMDs appears to be well conserved with *H. sapiens*, however, they are not as conserved as the TMDs of Grin1 subunits. This supports the phylogenetic evidence that AMPAR subunits in *Aplysia* are closely related to *H. sapiens* subunits and may perform similar functions. In the phylogeny, kainate receptor subunits of the prostostomes were highly divergent from vertebrates, however the TMDs are a notable exception and appeared to be well-conserved with *H. sapiens* in this analysis.

### Evaluation of Grin2 genes in Aplysia genomic DNA

While a search of the recently revised *A. californica* genome revealed only one gene similar to Grin2 subunits in Chordata, the presence of gaps in this genome sequence raises the possibility that additional genes may have been missed. A Southern blot analysis of genomic DNA of *Aplysia* demonstrated only a single band homologous to a conserved region of Grin2 (data not shown). This band was at the expected molecular weight for the previously identified *Aplysia* Grin2, suggesting that no other Grin2 genes are present in this genome. This result was not surprising considering that there is only one Grin2 gene found in other Protostomia species [56].

### Quantification of iGluR gene expression in the nervous system

**Fig. 5:**
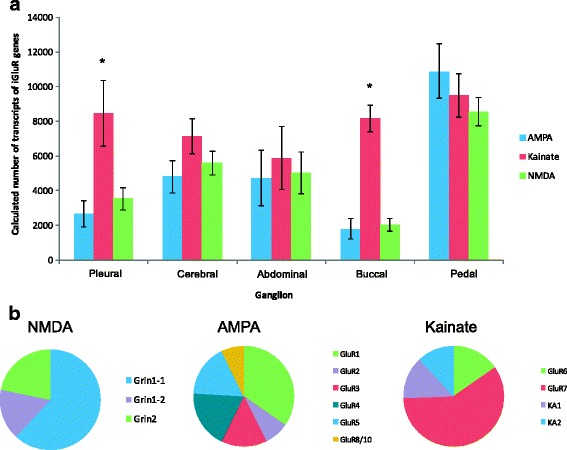
iGluR expression levels in *Aplysia* ganglia. The expression levels of each subtype of iGluR were determined by quantitative real-time PCR. Absolute copy number was calculated using standard curves. **a** Comparisons of total expression of each iGluR subtype in different *Aplysia* ganglia, with calculated number of transcripts on the y-axis. Total kainate receptor subunit expression was significantly greater than other subtypes in both the pleural and buccal ganglia (one-way ANOVA, Tukey’s post-hoc, *p* ≤ 0.05) (**b**) Pie charts showing each subunit’s contribution to the total expression of its subtype

The expression of identified iGluR in all *Aplysia* nervous system ganglia is shown in Fig. 5 (s﻿ee Additional file ﻿5 for Cts). All twelve subunits, as well as the splice variant Grin1-2, were expressed in all ganglia, with strong differences both within and between different iGluR subtypes. The subtype with the highest expression varied across the different ganglia (Fig. 5a). In most tissues the kainate-like receptor genes were expressed at the highest levels, followed by NMDAR-like genes, with AMPAR-like genes expressed at the lowest levels. The highest expression of all three iGluR subtypes was found in the pedal ganglion, suggesting that this ganglion has the greatest reliance on L-Glu-mediated neurotransmission. The buccal ganglion showed the lowest expression for both NMDAR and AMPAR genes and showed significantly greater (*p* ≤ 0.05) expression of kainate receptor subunits.

Expression of NMDAR genes was primarily due to high expression of Grin1-1, representing >60% of NMDAR expression in nearly all nervous system tissues (Fig. 5b). These data suggest that Grin1 may be the subunit that contributes the most to NMDAR-mediated physiological responses to L-Glu in the nervous system of *Aplysia*. The lone exception was the buccal ganglion, where the splice variant Grin1-2 was the most highly expressed NMDAR subunit gene in the majority of the six animals studied. A similar pattern of high expression of one gene was observed with kainate receptor genes, where the GluR7 subunit comprised the majority of this subtype’s gene expression. The distribution of expression of AMPAR subunits favored no single subunit gene dominating the expression of this subtype.

## Discussion

iGluR mediated responses in the nervous system are of particular interest due to their role in synaptic plasticity associated with learning and memory. Protostomes have a long history of use as model organisms for studies of iGluR mediated synaptic plasticity due to their simple nervous systems and well-defined neural circuits. Many plasticity-related changes in the vertebrate nervous system were first discovered in protostomes, and were subsequently shown to be conserved in chordates [9]. Further studies in vertebrates have identified the NMDAR and AMPAR subtypes of iGluR underlying these processes. Despite the utility of protostomes for iGluR mediated responses in the nervous system, identification of iGluR subunits that are orthologous to chordate iGluR has so far not been thoroughly studied. The application of phylogenetic methods to specific protein families is a useful procedure to shed light on their functional status.

We conducted a phylogenetic analysis of iGluR spanning all three superclades of bilaterians; Deuterostomia (including Chordata), and their sisterclade Protostomia, consisting of Ecdysozoa and Lophotrochozoa. We found that the evolutionary history of iGluR in Bilateria is distinct for each iGluR subtype. It was possible to identify protostome iGluR subunits most closely related to chordate iGluR, with possibly similar functional roles, for both NMDAR and AMPAR genes.

The best supported conclusion from the analysis presented is that clear orthologous groups are formed for both Grin1 and Grin2 genes. This is an extension of the previous report that protostome Grin1 and Grin2, including *Aplysia*, are more closely related to chordate Grin1 and Grin2, respectively, than to each other [35] and further supports the close kinship of all known bilaterian NMDAR subunits. This study confirms an ancient duplication that generated two orthologous NMDAR genes present in the common bilaterian ancestor. Both orthogroups have been under high selective constraint that has maintained the function of these genes at least since the divergence of deuterostomes and protostomes, thus making protostome model organisms excellent study systems for NMDAR.

The number of NMDAR subunits varies greatly between deuterostomes and protostomes. Chordate species in this study express 3–8 Grin1 splice variants, while only 1 Grin1 splice variant was identified in *Aplysia*. The chordates also express a greater number of Grin2 genes and splice variants. Two independent rounds of whole genome duplication in the deuterostome lineage since its split from the bilaterian ancestor, that also gave rise to Lophotrochozoa [44], are hypothesized to be the origin of these duplicated Grin2 genes [56, 57]. The regulatory C-terminus contains most of the sequence variability in different vertebrate Grin2 genes. We hypothesize that the retention of duplicated Grin2 genes is explained by the subfunctionalization model, whereby each duplicate gene copy maintains a subset of its original function [58].

The NMDAR subunits involved in receptor assembly have been shown to strongly alter pharmacological and biophysical properties of the channel, including sensitivity to allosteric modulators, single channel conductance, and activation/deactivation kinetics [59–61]. The greater number of NMDAR subunits available for complete receptors in chordates increases the different physiological responses possible, and likely underlies the more complex and nuanced signaling capability observed in vertebrates. Furthermore, activation of NMDAR has been shown to be an essential component of synaptic plasticity and memory formation in vertebrates [62], and knockdown of NMDAR can disrupt learning in protostome species [24, 63, 64]. In spite of the greater diversity of NMDAR subunits in chordates, homology of Grin1 and Grin2 across bilaterian animals suggests that a wealth of discovery in the mechanisms of NMDAR-induced plasticity is nevertheless possible in simpler nervous systems such as *Aplysia*, with relevance to vertebrate NMDAR physiology, including that of humans.

AMPAR subtype genes also form an orthologous group, with evidence of a single ancestral AMPAR gene in the common bilaterian ancestor. Unlike NMDAR, the number of protostome genes in this orthogroup is highly variable and has been subject to extensive lineage and taxon specific gene duplications. For example, in the Ecdysozoan superclade, *Pripaulus*, *Drosophila*, and *C. elegans* each have two inparalogous AMPAR genes most closely related to genes in their own species, suggesting that each gene is a paralog that arose independently in each lineage. Interestingly, the other two Ecdysozoan species studied, *Daphnia* and *Tribolium*, do not have any genes within the AMPAR orthogroup. In Lophotrochozoans expansion of AMPAR genes is particularly prevalent in *Aplysia*, where multiple gene duplication events have resulted in six AMPAR genes.

Whether AMPAR physiology in protostomes will bear the same resemblance to chordate AMPAR, as is the case with NMDAR, is uncertain. The name AMPAR for this subtype is likely inaccurate in protostomes. Isolated *Aplysia* sensory neurons do not respond to exogenously applied AMPA, and AMPAR antagonists do not block whole cell currents elicited by the L-Glu analog D-aspartate (D-Asp; [55]). Yet *Aplysia* AMPAR have physiological roles with relevance to vertebrate learning, with AMPAR antagonists inhibiting facilitation [65, 66]. Synaptic plasticity in vertebrates is also AMPAR-dependent [67, 68]. AMPAR antagonists such as 6,7-dinitroquinoxaline-2,3-dione disodium (DNQX) and 6-cyano-7-nitroquinoxaline-2,3-dione disodium (CNQX) act on sites that are different than the agonist binding site. Similarity to chordate AMPAR suggests that *Aplysia* and other protostome AMPAR may bind agonists and antagonists differently than do those of vertebrates, yet operate similarly physiologically once activated.

Extensive gene gain and loss of AMPAR suggests less functional constraint compared to NMDAR genes, and may underlie these dissimilarities in AMPAR physiology in different taxonomic groups. Different numbers of AMPAR genes in different protostomes may result in unique receptor assemblages conveying different physiological properties in each taxon, thus the exact role of AMPAR may be unique in different lineages.

Kainate receptors are the least conserved subtype, with no strongly supported monophyletic group, but were clearly present in the common bilaterian ancestor. Kainate receptors play a minor role in synaptic signaling [69], and are not believed to be as involved in learning and memory as NMDAR and AMPAR [70]. Therefore, it is unsurprising that there may be less functional constraint on these receptors than there is on AMPAR and NMDAR. Large divergence of kainate receptor genes makes it difficult to predict the functional relevance of these genes to iGluR mediated excitability in the nervous system of protostomes.

In agreement with previous studies, vertebrate iGluR subunits were arranged into three distinct clades corresponding to the three different agonists [6]. Our analysis of *Aplysia* only iGluR genes indicated that NMDAR and AMPAR subunits form well-defined clades and thus may be capable of forming functional iGluR channels. In contrast, the makeup of kainate receptors is less clear, due to the lack of monophyletic groups in the protostomes.

All 12 identified iGluR subunits were expressed in all *Aplysia* ganglia, extending the results of an *in situ* hybridization study concluding that Grin1 was expressed throughout the nervous system [35]. This attests to the importance of L-Glu mediated fast synaptic transmission in all parts of the *Aplysia* nervous system. Grin1 expression was highest in nearly all *Aplysia* ganglia, with its two splice variants Grin1-1 and Grin1-2 together comprising ~75% of total NMDAR expression. A recent study using a nuclease protection assay showed that expression of Grin1 comprised 67–88% of the total Grin expression in rat brain [71]. When combined with homology discussed earlier, this suggests that the regulation and function of NMDAR in *Aplysia* are highly conserved with those of vertebrates.

Variable and spatially distinct expression of the three iGluR subtypes was observed in the ganglia of the *Aplysia* brain. Pedal ganglion had the highest iGluR expression, with the glutamatergic nature of pedal motoneuron transmission corroborated by physiological studies [72, 73]. Variations in the frequency and amplitude of ionic currents activated by the iGluR agonists L-Glu and D-Asp have been documented in neurons isolated from different ganglia [74, 75], lending support to the non-uniformity of the receptor expression patterns. Studies in mammalian brains have shown both spatial and developmental variations in patterns of expression of NMDAR and AMPAR subunits, with some subunits specific for certain brain regions, or variable expression dependent on the stage of development [76, 77].

To place bilaterian iGluR into a larger evolutionary perspective, insights about the deep origins of iGluR have recently emerged from discoveries on ctenophores. Studies of candidate iGluR genes in the ctenophore *Mnemiopsis leidyi,* which is currently thought basal to bilaterian animals [78], revealed that ctenophore iGluR form a monophyletic clade separate from, and ancestral to, chordates [79]. Thus it appears that subunit types emerged after the ctenophores split, but before the divergence of Deuterostomia, Ecdysozoa, and Lophotrochozoa. This is suggestive of individual AMPAR and NMDAR subunits evolving prior to the last common bilaterian ancestor, but after divergence from ctenophores. Furthermore, two studies using expressed sequence tags (EST), genome organization, gene structure and functional content found lower amino acid substitution rates in Lophotrochozoa than Ecdysozoa relative to chordates. These findings suggest that genes in Lophotrochozoa are more likely to have greater sequence similarity to chordates than Ecdysozoan genes, and hence may be more likely to be functional equivalent to chordates.

NMDAR and AMPAR subtypes of iGluR are vital to synaptic plasticity associated with vertebrate learning. This study confirms the ancestral origins of NMDAR and AMPAR genes and also, but less strongly supported, ancestral kainate receptor genes in Bilateria. These findings underscore the utility of *Aplysia* and other protostome models for the studies of AMPAR and NMDAR mediated responses in the nervous system.

## Conclusions

This is the first analysis of the phylogenetic relationships between subtypes of iGluR genes across Bilateria. For decades, model organisms from the Protostomia have been used as models of nervous system function, and we show that AMPAR and NMDAR subtypes were present in the common bilaterian ancestor and have been maintained as orthologous groups. Functional constraint preventing amino acid substitutions in pore regions of NMDAR suggests a highly conserved function of these subunits and potentially a conserved mechanism of learning. Kainate receptor subunits are the least conserved and may not play the same role in protostomes and deuterostomes. qPCR results demonstrate that iGluR are expressed ubiquitously throughout the nervous system of *Aplysia*, underscoring the importance of this model to understanding iGluR mediated nervous system function.

## Additional files

Additional file 1: iGluR protein accession numbers. Complete list of protein accession numbers of all iGluR proteins used to create phylogenies. (XLSX 35 kb)
Additional file 2: qPCR Primer Pairs. List of forward and reverse primers used to amplify unique qPCR products for each *Aplysia* iGluR gene and the Grin1-2 splice variant. All amplicons were 100–150bp in length. (XLSX 53 kb)
Additional file 3: Protein alignments used to create phylogenetic trees. Protein alignments both before and after sequence trimming with trimAl that were used to create each phylogeny, including: full iGluR phylogeny, NMDAR, AMPAR, and kainate receptor trees, and *Aplysia* only iGluR tree. (ZIP 292 kb)
Additional file 4: Additional phylogenetic trees and hydrophobicity plots. Additional phylogenies were created using trimmed alignments from Additional file 3. **Figure S1**: *Aplysia* only phylogeny inferred by the maximum likelihood method to identify subunits that form monophyletic clades in *Aplysia* and thus may form complete receptors. NMDAR and AMPAR subunits form monophyletic clades, while kainate receptor subunits did not form a clade. **Figure S2**: NMDAR only subtype tree using sequences identified as NMDAR in the full phylogeny. Monophyletic relationships are formed between each protostome and chordate NMDAR subunit, as observed in the full phylogeny. **Figure S3**: AMPAR subtype only tree indicates a single AMPAR gene in the common bilaterian ancestor. **Figure S4**: Kainate receptor subunits from chordates and protostomes do not form a strongly supported clade, unlike the strongly support clades observed in the NMDAR and AMPAR subtypes. **Figure S5**: Hydrophobicity plot of *H. sapiens* GRIA1 and *Aplysia* GluR1 TMDs. Similarity in TMD1 and TMD3 between the sequences suggest similar ion channel structure. **Figure S6**: Hydrophobicity plot of *H. sapiens* GRIK1 and *Aplysia* GluR7 shows high conservation of TMDs. (PDF 131 kb)
Additional file 5: Average Ct values from qPCR. Average Ct values of two technical replicates for each iGluR gene in all nervous system ganglia of *Aplysia* used to calculate copy numbers for each subunit. (XLSX 43 kb)

## Abbreviations

2R: 2 round genome duplication
ABD: Agonist binding domain
AMPAR: α-Amino-3-hydroxy-5-methyl-4-isoxazolepropionic acid receptor
ASW: Artificial sea water
CNQX: 6-cyano-7-nitroquinoxaline-2,3-dione disodium
CNS: Central nervous system
Ct: Cycle threshold
D-Asp: D-aspartate
dCTP: Deoxycytidine triphosphate
DNQX: 6,7-dinitroquinoxaline-2,3-dione disodium
EST: Expressed sequence tag
GRIA: Glutamate Receptor Ionotropic AMPA
GRIK: Glutamate Receptor Ionotropic Kainate
Grin: Glutamate Receptor Ionotropic NMDA
iGluR: Ionotropic L-Glu receptor
L-Glu: L-Glutamate
mGluR: Metabotropic L-Glutamate receptor
mRNA: Messenger RNA
MUSCLE: Multiple sequence comparison by log-expectation
NMDAR: N-methyl-D-aspartate receptor
NTD: N-terminal domain
PCR: Polymerase chain reaction
qPCR: Quantitative polymerase chain reaction
SDS: Sodium dodecyl sulphate
SSC: Sodium citrate buffer
TMD: Transmembrane domain
