# Supplementary material for: Phylogenetic analysis of ionotropic L-glutamate receptor genes in the Bilateria, with special notes on Aplysia californica
Source: BMC Evol Biol. 2017 Jan 11;17:11. doi: 10.1186/s12862-016-0871-1 (PMC5225553; doi:10.1186/s12862-016-0871-1)
Supplement: Additional file 4: — Additional phylogenetic trees and hydrophobicity plots. Additional phylogenies were created using trimmed alignments from Additional file 3. Figure S1: Aplysia only phylogeny inferred by the maximum likelihood method to identify subunits that form monophyletic clades in Aplysia and thus may form complete receptors. NMDAR and AMPAR subunits form monophyletic clades, while kainate receptor subunits did not form a clade. Figure S2: NMDAR only subtype tree using sequences identified as NMDAR in the full phylogeny. Monophyletic relationships are formed between each protostome and chordate NMDAR subunit, as observed in the full phylogeny. Figure S3: AMPAR subtype only tree indicates a single AMPAR gene in the common bilaterian ancestor. Figure S4: Kainate receptor subunits from chordates and protostomes do not form a strongly supported clade, unlike the strongly support clades observed in the NMDAR and AMPAR subtypes. Figure S5: Hydrophobicity plot of H. sapiens GRIA1 and Aplysia GluR1 TMDs. Similarity in TMD1 and TMD3 between the sequences suggest similar ion channel structure. Figure S6: Hydrophobicity plot of H. sapiens GRIK1 and Aplysia GluR7 shows high conservation of TMDs. (PDF 131 kb) [file 12862_2016_871_MOESM4_ESM.pdf]

Page	Figure
2	**Fig. S1. Aplysia iGluR protein tree**.
3	**Fig. S2. NMDAR subtype only tree**
4	**Fig. S3. AMPAR subtype only tree**
5	**Fig. S4. Kainate receptor subtype only tree**
6	**Fig. S5. Representative AMPA subunit TMD Hydrophobicity**
7	**Fig. S6. Representative kainate subunit TMD Hydrophobicity**

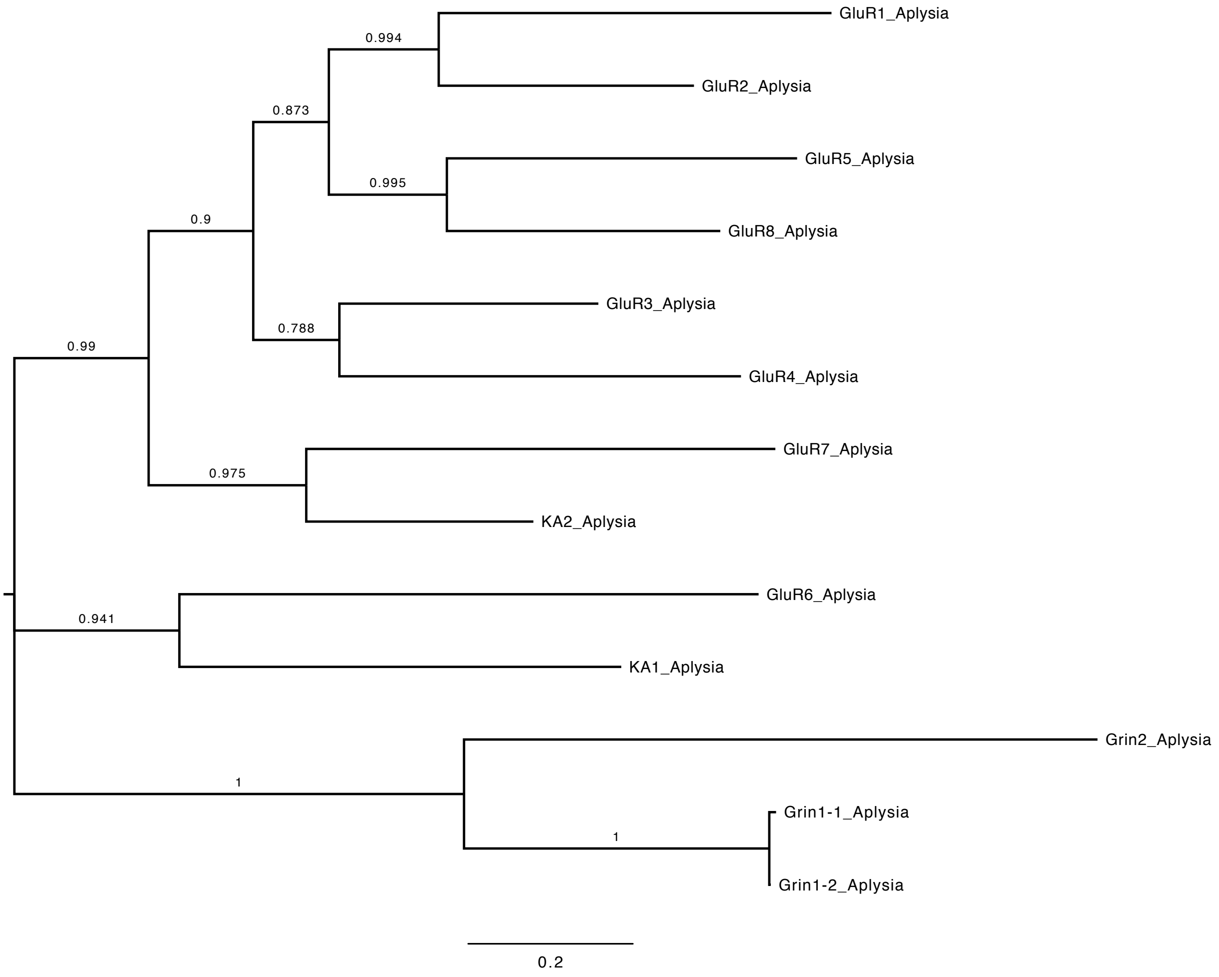

Fig. S1. Aplysia iGluR protein tree. Subunits within a clade can form functional receptors in vertebrates, and clades formed by both AMPAR and NMDAR-type subunits of *Aplysia* suggest subunits within these clades may form complete receptors. Kainate receptor subunits do not form a clade. GluR7 and KA2 are the more likely kainate receptor subunit candidates based on their positions full tree. Numbers indicate bootstrap support and the scale bar represents 0.2 substitutions per site.

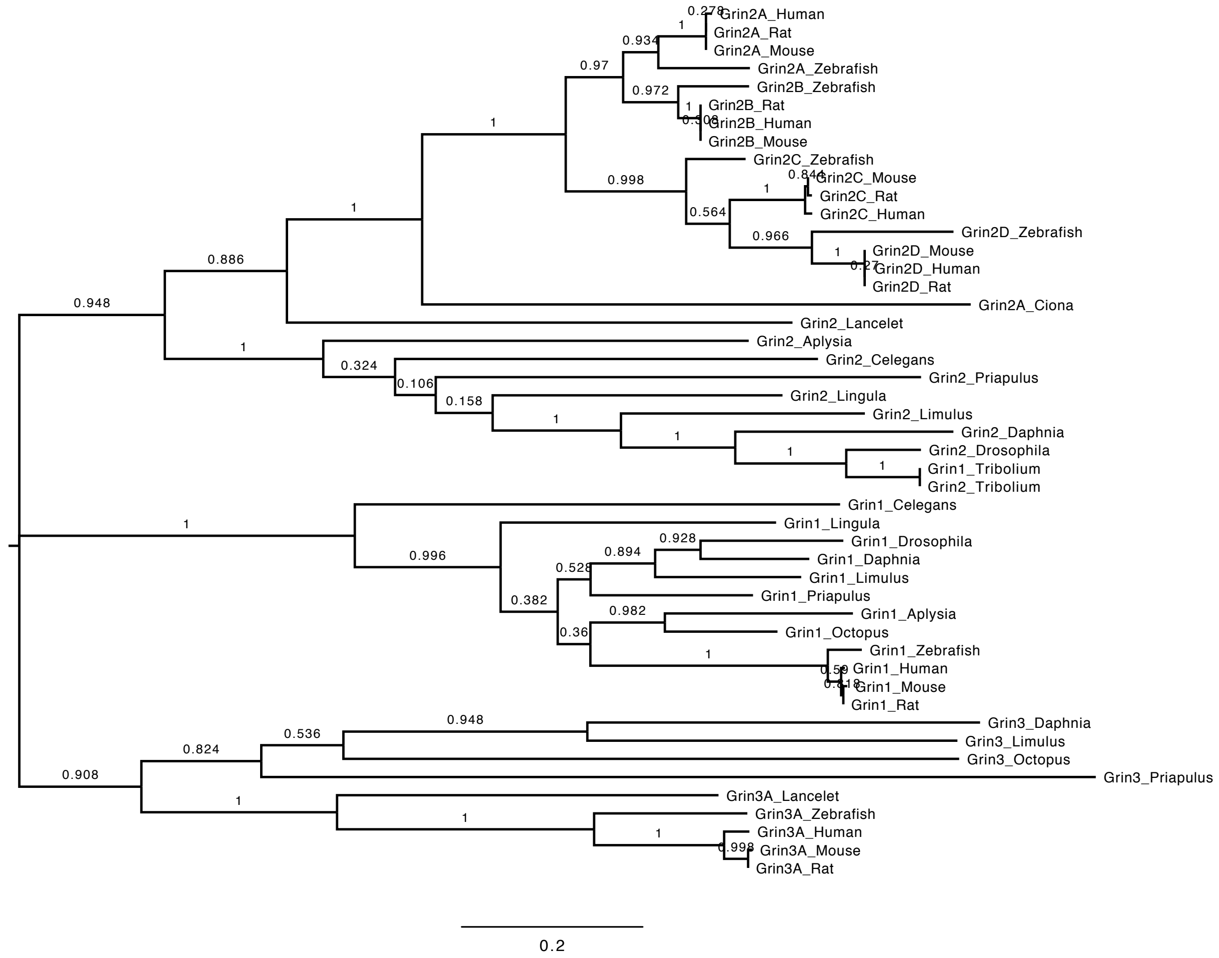

Fig. S2. NMDAR subtype only tree. Phylogeny using sequences identified in the full phylogeny as NMDAR. Protostome sequences show monophyletic relationships with chordates for each subunit as seen in the full phylogeny. After alignment trimming there were 467 positions used for analysis. Numbers indicated bootstrap support and the scale bar represent 0.2 substitutions per site.

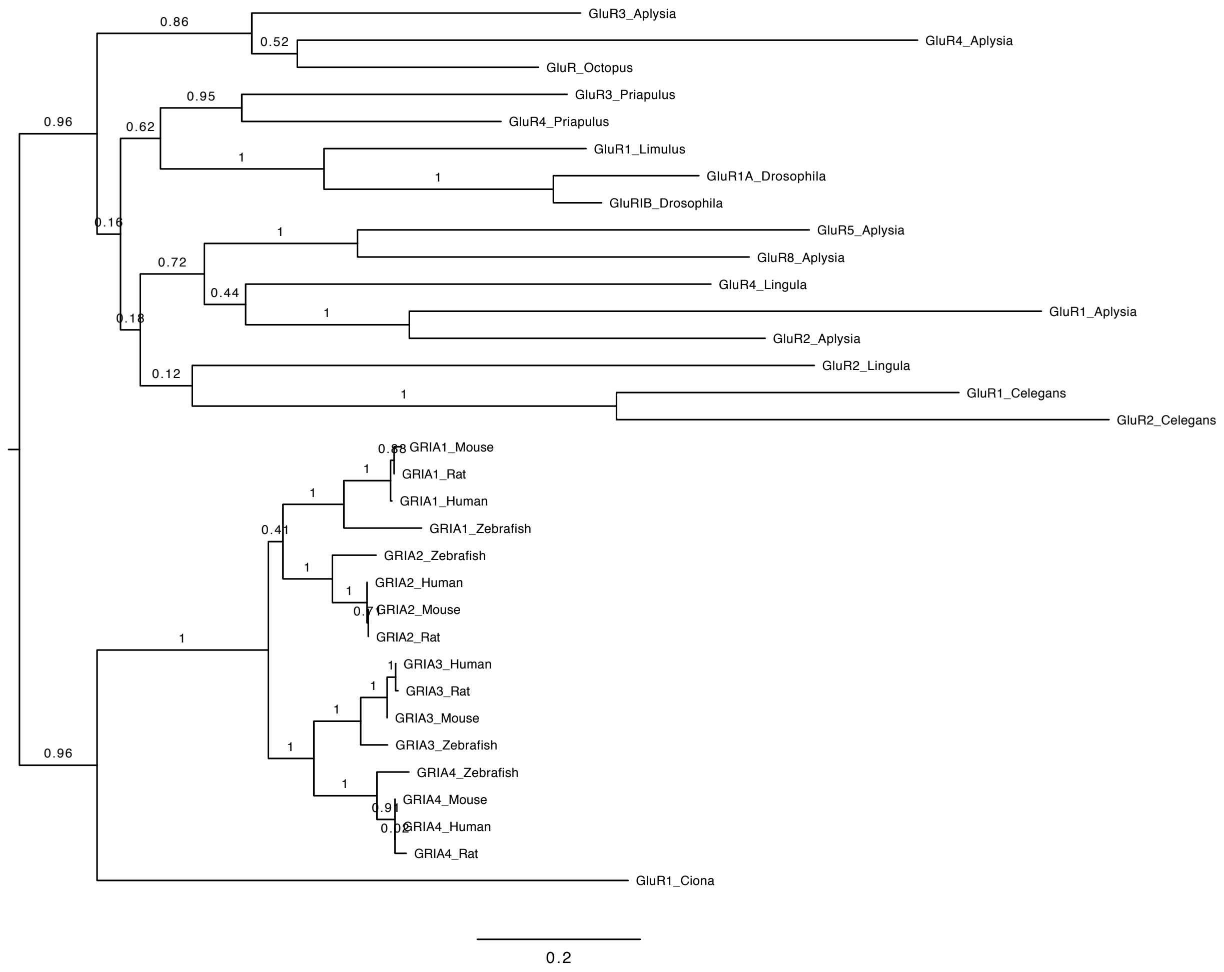

Fig. S3. AMPAR subtype only tree. Phylogeny of sequences identified as AMPAR genes in the full phylogeny. The tree indicates a single AMPAR gene in the common bilaterian ancestor as seen in the full phylogeny. After alignment trimming there were 665 positions used for analysis. Numbers indicated bootstrap support and the scale bar represent 0.2 substitutions per site.

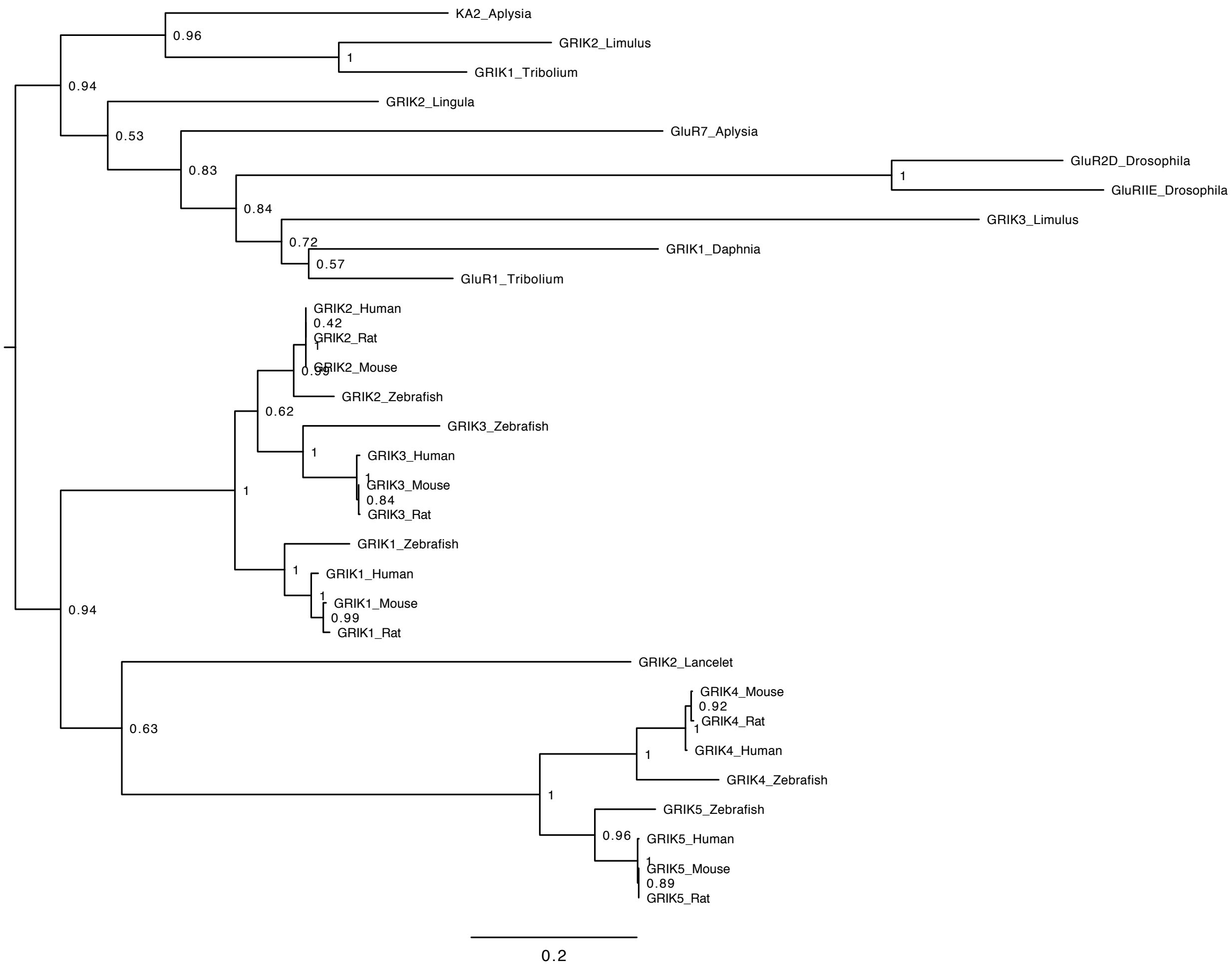

Fig. S4. Kainate receptor subtype only tree. Phylogeny of sequences identified as kainate receptor genes in the full tree. There were 687 positions in the alignment after trimming. Scale bar represents 0.2 amino acid substitutions per site.

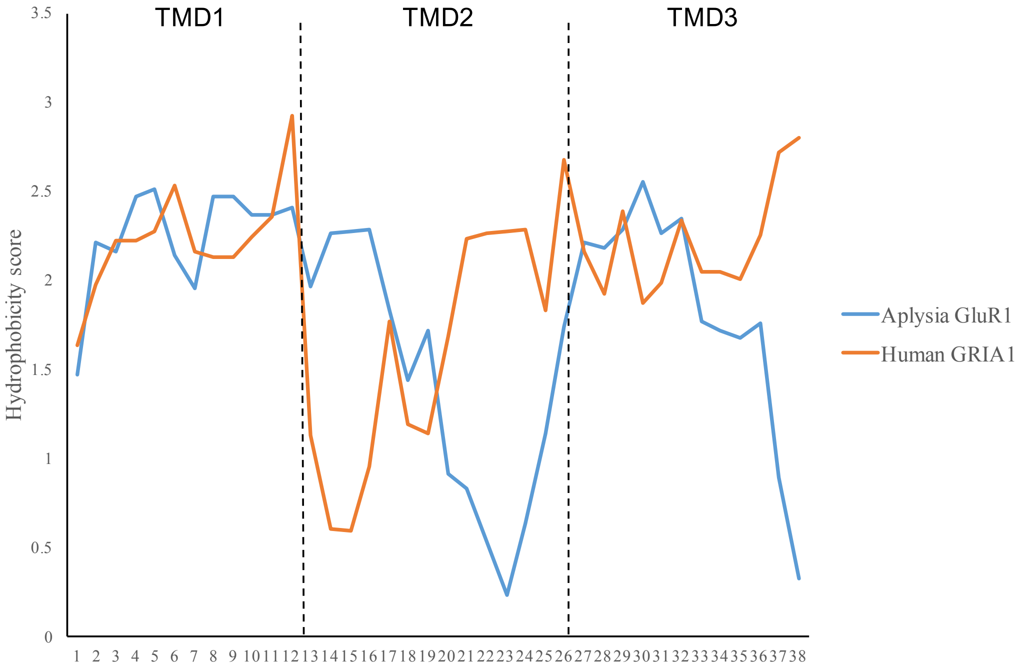

Fig. S5. Representative AMPA subunit TMD Hydrophobicity. *Aplysia* GluR1 is monophyletic with vertebrate AMPAR subunits in the phylogeny, and shows similar hydrophobicity in its TMD1 and TMD3 to Human GRIA1. This suggests similarities in the protein structure of the ion channel of these two subunits.

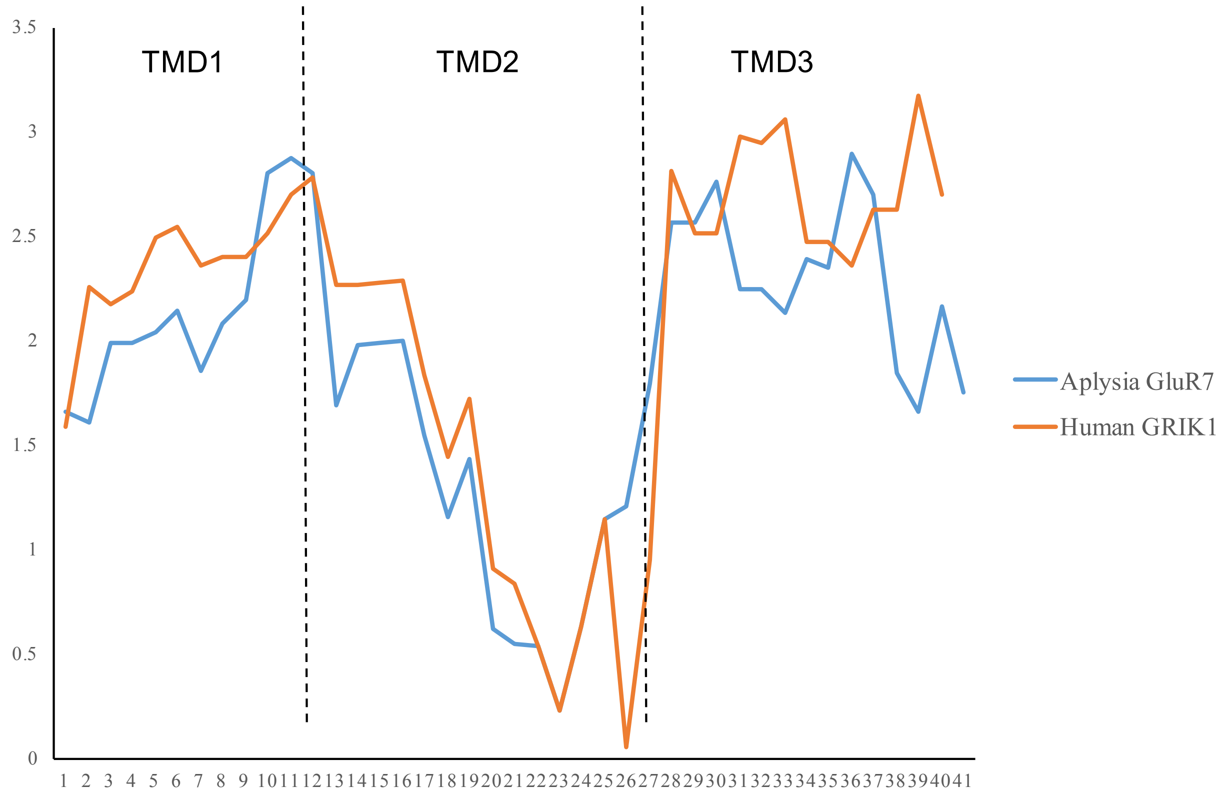

Fig. S6. Representative kainate subunit TMD Hydrophobicity. *Aplysia* GluR7 is monophyletic with vertebrate kainate receptor subunits in the phylogeny, and shows similar hydrophobicity in its TMDs to Human GRIK1. This suggests similarities in the protein structure of the ion channel of these two subunits.
